# Identification of parameter correlations for parameter estimation in dynamic biological models

**DOI:** 10.1186/1752-0509-7-91

**Published:** 2013-09-22

**Authors:** Pu Li, Quoc Dong Vu

**Affiliations:** 1 Department of Simulation and Optimal Processes, Institute of Automation and Systems Engineering, Ilmenau University of Technology, P. O. Box 100565, 98684 Ilmenau, Germany

**Keywords:** Parameter estimation, Identifiability, Parameter correlation, Data sets with different inputs, Zero residual surfaces, Experimental design

## Abstract

**Background:**

One of the challenging tasks in systems biology is parameter estimation in nonlinear dynamic models. A biological model usually contains a large number of correlated parameters leading to non-identifiability problems. Although many approaches have been developed to address both structural and practical non-identifiability problems, very few studies have been made to systematically investigate parameter correlations.

**Results:**

In this study we present an approach that is able to identify both pairwise parameter correlations and higher order interrelationships among parameters in nonlinear dynamic models. Correlations are interpreted as surfaces in the subspaces of correlated parameters. Based on the correlation information obtained in this way both structural and practical non-identifiability can be clarified. Moreover, it can be concluded from the correlation analysis that a minimum number of data sets with different inputs for experimental design are needed to relieve the parameter correlations, which corresponds to the maximum number of correlated parameters among the correlation groups.

**Conclusions:**

The information of pairwise and higher order interrelationships among parameters in biological models gives a deeper insight into the cause of non-identifiability problems. The result of our correlation analysis provides a necessary condition for experimental design in order to acquire suitable measurement data for unique parameter estimation.

## Background

Parameter estimation of dynamic biological models described by nonlinear ordinary differential equations (ODEs) poses a critical challenge. A special feature of biological models is that they usually contain a large number of parameters among which correlations exist [1,2]. In general, the quality of estimation results depends on the quality of data acquisition, the quality of the fitting method, and the quality of the model. Good experiment design can lead to highly informative data which will enhance the accuracy and identifiability of model parameters. Therefore, the task of parameter estimation demands an interactive endeavour of experiment and computation [3,4].

To fit parameters to measured data a numerical method for solving an optimization problem is required. Available methods for carrying out this task can be classified into deterministic approaches (e.g., multiple shooting [5,6], collocation on finite elements [7], global approaches [8,9]) and stochastic approaches (e.g. simulated annealing [10], genetic algorithms [11], and scatter search [12]). Using these approaches, model parameters can be well fitted to measured time courses provided from either experiment (*in vivo*) or simulation (*in silico*), i.e. high quality fits with minimal residual values can be obtained by global optimization methods.

However, even such a good fitting cannot guarantee unique parameter estimation, due to correlations among the parameters. The correlation phenomenon can be explained by the biological background, e.g. genes or proteins which are expressed in a correlated manner, correlations of expression levels between cells. As a consequence, certain regions in the parameter space correspond to good model predictions. It means that the residual value (quadratic error) remains low even if the parameters vary in certain regions. By testing 17 biological models, Gutenkunst et al. [13] concluded that collective fits to large amounts of ideal time-series data lead to the fact that some eigenvectors are orders of magnitudes better constrained than others.

Correlated parameters are non-identifiable. If the non-identifiability does not change for any data, these parameters are called structurally non-identifiable. On the contrary, if the non-identifiability can be remedied by data improvement, they are practically non-identifiable [14,15]. Identifiability analysis represents an important ongoing topic in the literature which can be in general categorized into two major groups: *a priori* and *a posteriori* methods [1,16]. Without any requirement of measurement data, global (structural) identifiability can be determined by *a priori* methods [17-19]. Since these methods are normally based on differential algebra, their application to high dimensional complex models can be limited.

The *a posteriori* methods reveal practical identifiability properties based on results from fitting parameters to available data sets. In most studies, correlations are detected by analysing the sensitivity matrix and the Fisher information matrix (FIM) [1,16,20-23], from which local confidence regions of parameter solutions can be obtained. Sensitivity analysis is well suitable to linear models but will have limitations for highly nonlinear models [14,24].

Recently, Raue et al. [15] proposed to use profile likelihood to detect non-identifiability for partially observable models. The parameter space is explored for each parameter by repeatedly fitting the model to a given data set, which then provides a likelihood-based confidence region for each parameter. Results from this method show that the number of practically non-identifiable parameters will decrease when more data sets are used [25].

An aim of identifiability analysis is to determine if the parameters of a model are identifiable or not, i.e. whether its parameters can be uniquely estimated. The profile likelihood approach can also offer information on the correlated relations among the parameters [15,25-27]. The information on parameter correlations (e.g. correlated groups, correlated forms in a group etc.) is important for experimental design, so that a series of experimental runs with determined conditions can be carried out to acquire proper measurement data sets for improving the quality of parameter estimation.

Very few studies have been made to investigate parameter correlations in biological models. Yao et al. [21] used the rank of the sensitivity matrix to determine the number of estimable parameters. However, the subsets of correlated parameters cannot be identified based on this result. Chu and Hahn [28] proposed to check the parallel columns in the sensitivity matrix to determine parameter subsets in which the parameters are pairwise correlated. Quaiser and Mönnigmann [29] proposed a method to rank the parameters from least estimable to most estimable. These methods, however, cannot identify parameter groups in which more than two parameters are correlated together but not in pairwise, i.e. the corresponding columns in the sensitivity matrix are linearly dependent but not parallel. Such correlations are called higher order interrelationships among parameters [16].

In this paper, “parameter correlations” means a group of parameters in the model equations which are mathematically related to each other through some implicit functions, i.e. among the parameters there is a functional relationship [15,26,27]. Correlated parameters will be structurally non-identifiable, if the functional relationship does not depend on the control variables which determine experimental conditions and thus measured data. On the other hand, they will be practically non-identifiable, if the functional relationship depends on the control variables.

In this paper, we present an approach which is able to identify both pairwise and higher order parameter correlations. Our approach is based on analysis of linear dependences of the first order partial derivative functions of model equations. In a given model there may be a number of groups with different number of correlated parameters. We propose to identify these groups by analysing the correlations of the columns of the state sensitivity matrix which can be derived directly from the right-hand side of the ODEs. Therefore, the method proposed in this paper is *a priori* in nature, which means that the parameter correlations considered in this paper are not from the results of data-based estimation. A geometric interpretation of parameter correlations is also presented. Using this approach, groups of correlated parameters and the types of correlations can be identified and, hence, the parameter identifiability issue can be addressed. Moreover, the relationship between parameter correlations and the control inputs can be derived. As a result, both structural and practical non-identifiabilities can be identified by the proposed approach.

In the case of practical non-identifiability, the parameter correlations can be relieved by specifying the values of control inputs for experimental design. Based on the correlation analysis, the maximum number of parameters among the correlation groups can be determined, which corresponds to the minimum number of data sets with different inputs required for uniquely estimating the parameters of the model under consideration. Numerical results of parameter estimation of a three-step-pathway model clearly demonstrate the efficacy of the proposed approach.

## Methods

### Identification of parameter correlations

We consider nonlinear model systems described by

(1) $$\dot{x}\left(t\right)=f\left(x\left(t\right),u\left(t\right),p\right)$$

(2) $$y\left(t\right)=h\left(x\left(t\right),u\left(t\right),q\right)$$

where **x**(*t*) ∈ *R*^*n*^ is the state vector, **u**(*t*) ∈ *R*^*m*^ the control vector, and **y**(*t*) ∈ *R*^*r*^ the output vector, respectively. In this study, two different sets of parameters, i.e. **p** ∈ *R*^*NP*^ in the state equations and **q** ∈ *R*^*NQ*^ in the output equations, are considered. In most cases the number of parameters in the state equations is much larger than that in the output equations. Since the correlations of the parameters in the output Equation (2) are easier to identify, we concentrate on the analysis and identification of correlations of the parameters in the state Equation (1).

The equation of the state sensitivity matrix can be derived by taking the first order partial derivative of Eq. (1) with respect to parameters **p**

(3) $$\dot{S}=\left(\frac{\partial f}{\partial x}\right)S+\left(\frac{\partial f}{\partial p}\right)$$

where $S=\frac{\partial x}{\partial p}$ is the state sensitivity matrix. By solving this equation (see Additional file 1 for details) the state sensitivity matrix can be written as

(4) $$S=\int_{t_{0}}^{t}\left(V\left(\tau \right)\left(\frac{\partial f}{\partial p}\right)\right)\mathrm{d\tau}$$

where **V**(*τ*) is a matrix computed at time *τ*. It means that **S** has a linear integral relation with the matrix $\left(\frac{\partial f}{\partial p}\right)$ from *t*_0_ to *t*. If at any time $\left(\frac{\partial f}{\partial p}\right)$ has the same linearly dependent columns, the corresponding columns in **S** will also be linearly dependent, i.e. the corresponding parameters are correlated. Therefore, we can identify parameter correlations by checking the linear dependences of the column in the matrix $\left(\frac{\partial f}{\partial p}\right)$ which is composed of the first order partial derivatives of the right-hand side of the ODEs. Based on Eq. (2), the output sensitivity matrices are, respectively, given by

(5) $$\frac{\partial y}{\partial p}=\frac{\partial h}{\partial x}\frac{\partial x}{\partial p}=-\frac{\partial h}{\partial x}{\left(\frac{\partial f}{\partial x}\right)}^{-1}\frac{\partial f}{\partial p}$$

(6) $$\frac{\partial y}{\partial q}=\frac{\partial h}{\partial q}$$

To ensure unique estimation of the parameters (i.e. all parameters to be identifiable), based on the measured data of **y**, it is necessary that the columns in the output sensitivity matrices $\frac{\partial y}{\partial p},\;\frac{\partial y}{\partial q}$ are linearly independent. From Eq. (6), relations of the columns in $\frac{\partial y}{\partial q}$ can be easily detected. The difficulty comes from Eq. (5), since the sensitivity functions in $\frac{\partial y}{\partial p}$ cannot be analytically expressed. However, from Eq. (5), the output sensitivity matrix is a linear transformation of $\frac{\partial f}{\partial p}$. Consequently, there will be linearly dependent columns in $\frac{\partial y}{\partial p}$, if there are linearly dependent columns in $\frac{\partial f}{\partial p}$. It means the necessary condition for unique estimation of **p** is that, at least, the matrix $\frac{\partial f}{\partial p}$ must have a full rank. Based on Eq. (1), $\frac{\partial f}{\partial p}$ is expressed as vectors of the first order partial derivative functions

(7) $$\frac{\partial f}{\partial p}=\left[\frac{\partial f}{\partial p_{1}},\frac{\partial f}{\partial p_{2}},\cdots ,\frac{\partial f}{\partial p_{\mathrm{NP}}}\right]$$

Now we analyse relations between the partial derivative functions in Eq. (7). If there is no correlation among the parameters, the columns in Eq. (7) will be linearly *independent*, i.e. if

(8) $$\alpha_{1}\frac{\partial f}{\partial p_{1}}+\alpha_{2}\frac{\partial f}{\partial p_{2}}+\cdots +\alpha_{\mathrm{NP}}\frac{\partial f}{\partial p_{\mathrm{NP}}}=0$$

there must be *α*_*i*_ = 0, *i* = 1, ⋯, *NP*. Otherwise, there will be some groups of vectors in $\frac{\partial f}{\partial p}$ which lead to the following cases of linear dependences due to parameter correlations. Let us consider a subset of the parameters with *k* correlated parameters denoted as **p**_*sub*_ = [*p*_*s*+1_, *p*_*s*+2_, ⋯, *p*_*s*+*k*_]^*T*^ with *s* + *k* ≤ *NP*.

Case 1:

(9) $$\alpha_{1}\frac{\partial f}{\partial p_{s+1}}=\alpha_{2}\frac{\partial f}{\partial p_{s+2}}=\cdots =\alpha_{k}\frac{\partial f}{\partial p_{s+k}}$$

where *α*_*i*_ ≠ 0, *i* = 1, ⋯, *k*. Notice that the coefficient *α*_*i*_ may be a function of the parameters (i.e. *α*_*i*_(**p**)) and/or of control inputs (i.e. *α*_*i*_(**u**(*t*), **p**)). It should be also noted that the control inputs **u**(*t*) are considered as constants in these coefficients, since they will be specified in experimental design. The linear dependences described by Eq. (9) lead to pairwise correlations among the *k* parameters, i.e. any pair of the parameters in **p**_*sub*_ are correlated. Moreover, the correlations mean a functional relationship between the parameters, i.e. the relationship between the parameters can be expressed by an algebraic equation

(10) $$\phi_{\mathrm{sub}}\left(\gamma \left(p_{s+1},p_{s+2},\cdots ,p_{s+k}\right)\right)=0$$

where *γ*(*p*_*s*+1_, *p*_*s*+2_, ⋯, *p*_*s*+*k*_) denotes a function of the parameters with one set of pairwise correlated parameters. The parameters in this function are compensated each other in an algebraic relationship, e.g. *γ*(*p*_*s*+1_ + *p*_*s*+2_ + ⋯ + *p*_*s*+*k*_) or *γ*(*p*_*s*+1_*p*_*s*+2_⋯*p*_*s*+*k*_). Eq. (10) describes the functional relationship between the parameters, e.g. *ϕ*_*sub*_(*γ*( ⋅ )) = 1 + *γ*( ⋅ ) − (*γ*( ⋅ ))^2^ = 0. Due to the complexity of biological models, an explicit expression of this equation is not available in most cases.

If the coefficients in Eq. (9) are functions of only the parameters, i.e. *α*_*i*_(**p**), the parameters are *structurally* non-identifiable. In this case, the correlation relations in Eq. (9) will remain unchanged by specifying *any* values of control inputs. It means that the non-identifiability cannot be remedied through experimental design.

If the coefficients in Eq. (9) are functions of both the parameters and control inputs, i.e. *α*_*i*_(**u**, **p**), the parameters are *practically* non-identifiable. Different values for **u** can be specified which lead to different *α*_*i*_(**u**, **p**), such that Eq. (9) will not hold and therefore the parameter correlations will be relieved. Since *k* parameters are correlated, *k* different values of the control inputs **u**^(*j*)^, (*j* = 1, ⋯, *k*) are required, such that the matrix

(11) $$\frac{\partial f}{\partial p_{\mathrm{sub}}}=\left[\begin{array}{cccc} \frac{\partial f^{\left(1\right)}}{\partial p_{s+1}} & \frac{\partial f^{\left(1\right)}}{\partial p_{s+2}} & \cdots & \frac{\partial f^{\left(1\right)}}{\partial p_{s+k}}\\ \frac{\partial f^{\left(2\right)}}{\partial p_{s+1}} & \frac{\partial f^{\left(2\right)}}{\partial p_{s+2}} & \cdots & \frac{\partial f^{\left(2\right)}}{\partial p_{s+k}}\\ \vdots & \vdots & \cdots & \vdots \\ \frac{\partial f^{\left(k\right)}}{\partial p_{s+1}} & \frac{\partial f^{\left(k\right)}}{\partial p_{s+2}} & \cdots & \frac{\partial f^{\left(k\right)}}{\partial p_{s+k}} \end{array}\right]$$

has a full rank. Notice that the columns in Eq. (11) are only linearly dependent for the same input, but the columns of the whole matrix are linearly independent. In this way, the non-identifiability is remedied. Moreover, a suggestion for experimental design is provided for the specification of **u**^(*j*)^, (*j* = 1, ⋯, *k*) to obtain *k* distinct data sets which will be used for parameter estimation.

If all state variables are measurable, according to Eq. (4), this subset of parameters can be uniquely estimated based on the *k* data sets. If the outputs **y** are measured and used for the parameter estimation, it can be concluded from Eq. (5) that at least *k* data sets are required for unique parameter estimation.

Case 2:

(12) $$\begin{array}{l} \alpha_{1}\frac{\partial f}{\partial p_{s+1}}=\cdots =\alpha_{s+l_{1}}\frac{\partial f}{\partial p_{s+l_{1}}},\;\cdots ,\;\alpha_{s+l_{d-1}+1}\frac{\partial f}{\partial p_{s+l_{d-1}+1}}\\ \;=\cdots =\alpha_{s+k}\frac{\partial f}{\partial p_{s+k}} \end{array}$$

and

(13) $$\alpha_{s+k+1}\frac{\partial f}{\partial p_{s+1}}+\alpha_{s+k+2}\frac{\partial f}{\partial p_{s+l_{1}+1}}+\cdots +\alpha_{s+k+d}\frac{\partial f}{\partial p_{s+l_{d-1}+1}}=0$$

where *α*_*i*_ ≠ 0, *i* = 1, ⋯, *s* + *k* + *d*. Similarly, the coefficients may be functions of the parameters and/or of the control inputs. In this case, there are *d* sets of pairwise correlated parameters (Eq. (12)). A set is not correlated with another set, but all sets are correlated together (Eq. (13)). The functional relationship in this case can be expressed by

(14) $$\phi_{\mathrm{sub}}\left(\gamma_{1}\left(p_{s+1},\cdots ,p_{s+l_{1}}\right),\cdots ,\gamma_{d}\left(p_{s+l_{d-1}+1},\cdots ,p_{s+k}\right)\right)=0$$

Based on Eq. (12), the group with the maximum number of parameters max (*l*_1_, *l*_2_, ⋯, *l*_*d*_) is of importance for data acquisition. From Eq. (13), in the case of practical non-identifiability, data for at least *d* different inputs is required. The combination of Eqs. (12) and (13) leads to the conclusion that we need a number of max (*l*_1_, *l*_2_, ⋯, *l*_*d*_, *d*) data sets with different inputs to eliminate parameter correlations in this case.

Case 3:

(15) $$\alpha_{1}\frac{\partial f}{\partial p_{s+1}}+\alpha_{2}\frac{\partial f}{\partial p_{s+2}}+\alpha_{3}\frac{\partial f}{\partial p_{s+3}}+\cdots +\alpha_{k}\frac{\partial f}{\partial p_{s+k}}=0$$

where *α*_*i*_ ≠ 0, *i* = 1, ⋯, *k*. In this case, all *k* parameters are not pairwise correlated but they are correlated together in one group. The correlation equation in this case is expressed by

(16) $$\phi_{s}\left(p_{s+1},p_{s+1},\cdots ,p_{s+k}\right)=0$$

which means there is no set of correlated parameters in this case. The approach described above is able to identify pairwise and higher order parameter correlations in the state equations (Eq. (1)). In the same way, correlations among parameters in **q** in the output equations (Eq. (2)) can also be detected based on the first order partial derivative functions in Eq. (6).

From the results of this correlation analysis, the maximum number of correlated parameters of the correlation groups can be detected. This corresponds to the minimum number of data sets required for unique estimation of all parameters in the model. Furthermore, it is noted that the initial state of the model has no impact on the parameter correlations, which means that any initial state can be used for the experimental runs for the data acquisition.

For complex models, the correlation equations (Eqs. (10), (14), (16)) cannot be analytically expressed. A numerical method has to be used to illustrate the relationships of correlated parameters of a given model, which is discussed in the next section.

### Interpretation of parameter correlations

Here we give an interpretation of parameter correlations in a biological model. Geometrically, for *NP* parameters, i.e. **p** = [*p*_1_, *p*_2_, ⋯, *p*_*NP*_]^*T*^, the estimation task can be considered as searching for true parameter values p^*^ in the *NP*-dimensional parameter space. To do this, we need *NP* linearly independent surfaces in the parameter space which should pass through p^*^. Mathematically, such surfaces are described by linearly independent equations with the unknown parameters. We define such equations based on the results of fitting model Equations (1) to a data set (*j*) by minimizing the following cost function

(17) $$\min_{p}\;F^{\left(j\right)}\left(p\right)=\sum_{l=1}^{M}\sum_{i=1}^{n}w_{i,l}{\left(x_{i,l}^{\left(j\right)}\left(p\right)-{\hat{x}}_{i,l}^{\left(j\right)}\right)}^{2}$$

where *M* is the number of sampling points, *n* is the number of state variables and $\hat{x}$ denotes the measured data while **x**(**p**) the state variables predicted by the model. *w*_*i,l*_ are weighting factors. The fitting results will depend on the data set resulted from the control inputs **u**^(*j*)^, the values of *w*_*i,l*_, and the noise level of the measured data. For a geometric interpretation of parameter correlations, we assume to use idealized measurement data, i.e. data without any noises. Based on this assumption, the residual function (17) should be zero, when the true parameter set p^*^ is applied, i.e.

(18) $$x_{i,l}^{\left(j\right)}\left(p^{*}\right)-{\hat{x}}_{i,l}^{\left(j\right)}=0,\;i=1,\cdots ,n,\;l=1,\cdots ,M$$

It is noted that Eq. (18) is true for any noise-free data set employed for the fitting and independent of *w*_*i,q,*_. Now we define a zero residual equation (ZRE) as

(19) $$\varphi_{i,l}^{\left(j\right)}\left(p\right)=x_{i,l}^{\left(j\right)}\left(p\right)-{\hat{x}}_{i,l}^{\left(j\right)}=0$$

This equation contains the parameters as unknowns and corresponds to a zero residual surface passing through the true parameter point p^*^. It means that a zero residual surface is built by parameter values which lead to a zero residual value. This suggests that we can find p^*^ by solving *NP* linearly independent ZREs. From the first order Taylor expansion of Eq. (19), the linear dependences of ZREs can be detected by the columns in the following matrix

(20) $$\frac{\partial x^{\left(j\right)}}{\partial p}=\left[\frac{\partial x^{\left(j\right)}}{\partial p_{1}},\frac{\partial x^{\left(j\right)}}{\partial p_{2}},\cdots ,\frac{\partial x^{\left(j\right)}}{\partial p_{\mathrm{NP}}}\right]$$

where **x**^(*j*)^ = [*x*_1,1_^(*j*)^, *x*_1,2_^(*j*)^, ⋯, *x*_1,*M*_^(*j*)^, ⋯, *x*_*n*,1_^(*j*)^, *x*_*n*,2_^(*j*)^, ⋯, *x*_*n*,*M*_^(*j*)^]^*T*^. Eq. (20) is exactly the state sensitivity matrix calculated by fitting to the given data set (*j*). This means, under the idealized data assumption, a zero residual value delivered after the fitting is associated to surfaces passing through the true parameter point. When there are no parameter correlations, the number of linearly independent ZREs will be greater than *NP* and thus the true parameter point can be found by fitting the current data set.

If there are parameter correlations, the fitting will lead to zero residual surfaces in the subspace of the correlated parameters. For instance, for a group of *k* correlated parameters, the zero residual surfaces (Eq. (19)) will be reduced to a single ZRE represented by Eq. (10), Eq. (14), or Eq. (16). Therefore, in the case of *practical* non-identifiability, *k* data sets are needed to generate *k* linearly independent ZREs so that the *k* parameters can be uniquely estimated. In the case of *structural* non-identifiability, the correlated relations are independent of data sets. It means fitting different data sets will lead to the same ZRE and thus the same surfaces in the parameter subspace.

If the measured data are with noises, the fitting results will lead to a nonzero residual value and nonzero residual surfaces, i.e.

(21) $$\varphi_{i,l}^{\left(j\right)}\left(p\right)=x_{i,l}^{\left(j\right)}\left(p\right)-{\hat{x}}_{i,l}^{\left(j\right)}=\epsilon_{i,l}$$

where *ϵ*_*i*,*l*_ ≠ 0. Thus the nonzero residual surfaces will not pass through the true parameter point. However, based on Eq. (20) and Eq. (21) the first order partial derivatives remain unchanged. It means that parameter correlations do not depend on the quality of the measured data. Moreover, it can be seen from Eq. (19) and Eq. (21) that the zero residual surfaces and the nonzero residual surfaces will be parallel in the subspace of the correlated parameters.

## Results and discussion

We consider a three-step pathway modelled by 8 nonlinear ordinary differential equations (ODEs) containing 8 metabolic concentrations (state variables) and 36 parameters [30-32], as given in Eqs. (22-29). The *P* and *S* values in the ODEs are considered as two control inputs specified by experimental design. No output equations were considered for this model in the previous studies.

(22) $${\dot{x}}_{1}=\frac{p_{1}}{1+{\left(\frac{P}{p_{2}}\right)}^{p_{3}}+{\left(\frac{p_{4}}{S}\right)}^{p_{5}}}-p_{6}x_{1}$$

(23) $${\dot{x}}_{2}=\frac{p_{7}}{1+{\left(\frac{P}{p_{8}}\right)}^{p_{9}}+{\left(\frac{p_{10}}{x_{7}}\right)}^{p_{11}}}-p_{12}x_{2}$$

(24) $${\dot{x}}_{3}=\frac{p_{13}}{1+{\left(\frac{P}{p_{14}}\right)}^{p_{15}}+{\left(\frac{p_{16}}{x_{8}}\right)}^{p_{17}}}-p_{18}x_{3}$$

(25) $${\dot{x}}_{4}=\frac{p_{19}x_{1}}{p_{20}+x_{1}}-p_{21}x_{4}$$

(26) $${\dot{x}}_{5}=\frac{p_{22}x_{2}}{p_{23}+x_{2}}-p_{24}x_{5}$$

(27) $${\dot{x}}_{6}=\frac{p_{25}x_{3}}{p_{26}+x_{3}}-p_{27}x_{6}$$

(28) $${\dot{x}}_{7}=\frac{p_{28}x_{4}\left(S-x_{7}\right)}{p_{29}\left(1+\frac{S}{p_{29}}+\frac{x_{7}}{p_{30}}\right)}-\frac{p_{31}x_{5}\left(x_{7}-x_{8}\right)}{p_{32}\left(1+\frac{x_{7}}{p_{32}}+\frac{x_{8}}{p_{33}}\right)}$$

(29) $${\dot{x}}_{8}=\frac{p_{31}x_{5}\left(x_{7}-x_{8}\right)}{p_{32}\left(1+\frac{x_{7}}{p_{32}}+\frac{x_{8}}{p_{33}}\right)}-\frac{p_{34}x_{6}\left(x_{8}-P\right)}{p_{35}\left(1+\frac{x_{8}}{p_{35}}+\frac{P}{p_{36}}\right)}$$

This pathway model was studied by Moles et al. [31] using 16 noise-free data sets and Rodriguez-Fernandez et al. [32] using 16 both noise-free and noisy data sets, respectively. They showed some strong parameter correlations in several groups. Accurate parameter values were identified in [32]. However, a clear correlation analysis of the parameters and the relationship between the parameter correlations and the numbers of data sets with different inputs required for the parameter estimation were not given in the previous studies.

### Identification of correlations

Now we identify parameter correlations in this model using our approach. Given the model represented by Eqs. (22-29), the first order partial derivative functions can be readily derived leading to the following linear dependences (see Additional file 1 for detailed derivation).

From Eq. (22),

(30) $$\alpha_{1}\frac{\partial f_{1}}{\partial p_{1}}=\alpha_{2}\frac{\partial f_{1}}{\partial p_{2}}=\cdots =\alpha_{5}\frac{\partial f_{1}}{\partial p_{5}}$$

From Eq. (23),

(31) $$\alpha_{6}\frac{\partial f_{2}}{\partial p_{8}}=\frac{\partial f_{2}}{\partial p_{9}}\;\mathrm{and}\;\alpha_{7}\frac{\partial f_{2}}{\partial p_{7}}+\alpha_{8}\frac{\partial f_{2}}{\partial p_{10}}=\frac{\partial f_{2}}{\partial p_{8}}$$

From Eq. (24),

(32) $$\alpha_{9}\frac{\partial f_{3}}{\partial p_{14}}=\frac{\partial f_{3}}{\partial p_{15}}\;\mathrm{and}\;\alpha_{10}\frac{\partial f_{3}}{\partial p_{13}}+\alpha_{11}\frac{\partial f_{3}}{\partial p_{16}}=\frac{\partial f_{3}}{\partial p_{14}}$$

From Eq. (28),

(33) $$\alpha_{12}\frac{\partial f_{7}}{\partial p_{28}}+\alpha_{13}\frac{\partial f_{7}}{\partial p_{29}}=\frac{\partial f_{7}}{\partial p_{30}}$$

From Eq. (29),

(34) $$\alpha_{14}\frac{\partial f_{8}}{\partial p_{35}}=\frac{\partial f_{8}}{\partial p_{36}}\;$$

The coefficients in Eqs. (30) – (34), *α*_*i*_, (*i* = 1, ⋯, 14), are functions of the corresponding parameters and controls in the individual state equations (see Additional file 1). Based on these results, correlated parameters in this model can be described in 5 groups:

Group 1: *G*_1_(*p*_1_, *p*_2_, *p*_3_, *p*_4_, *p*_5_), among which any pair of parameters are pairwise correlated;

Group 2: *G*_2_(*p*_7_, *p*_8_, *p*_9_, *p*_10_), among which *p*_8_, *p*_9_ are pairwise correlated and *p*_7_, *p*_8_, *p*_10_ as well as *p*_7_, *p*_9_, *p*_10_ are correlated, respectively.

Group 3: *G*_3_(*p*_13_, *p*_14_, *p*_15_, *p*_16_), among which *p*_14_, *p*_15_ are pairwise correlated and *p*_13_, *p*_14_, *p*_16_ as well as *p*_13_, *p*_15_, *p*_16_ are correlated, respectively.

Group 4: *G*_4_(*p*_28_, *p*_29_, *p*_30_), the parameters are correlated together but not pairwise;

Group 5: *G*_5_(*p*_35_, *p*_36_), they are pairwise correlated.

Since the coefficients are functions of both of the parameters and the control inputs, these correlated parameters are practically non-identifiable for a single set of data. It is noted that, in *G*_2_ and *G*_3_, the maximum number of correlated parameters is three. Among the 5 correlated parameter groups the maximum number of correlated parameters is 5 (from *G*_1_). It means at least 5 data sets with different control values are required to uniquely estimate the 36 parameters of this model.

### Verification of the correlations by fitting the model

To verify the proposed approach and check the correlations in this model, we carried out numerical experiments by fitting the parameters to a certain number of simulated data sets with different inputs. The fitting method used is a modified sequential approach suitable for handling multiple data sets [33,34].

We used the nominal parameter values given in [31], initial state values as well as *P* and *S* values (see Additional file 1) given in [32] to generate 5 noise-free data sets with different inputs containing the time courses of the 8 state variables. For each data set 120 data points were taken with 1 minute as sampling time.

For fitting the parameters we used random values for all 36 parameters to initialize the computation and all weights in Eqn. (17) were set to 1.0. The results were taken by a threshold of the total residual value in the order of 10^-9^ when using noise-free data sets (see Table 1).

**Table 1:** Fitted parameter values based on different data sets

**No.**	P^*^	**P**^**(1)**^	**P**^**(1)+(2)**^	**P**^**(1)+(2)+(3)**^	**P**^**(1)+…+(4)**^	**P**^**(1)+…+(5)**^	**P**^**(1)+…+(5)**^ **(w)**
1(*G*_1_)	1.0	1.06763	1.07763	1.60486	1.73180	1.00000	0.97145
2(*G*_1_)	1.0	1.40146	0.91495	0.82116	0.75989	0.99998	1.05917
3(*G*_1_)	2.0	1.47116	1.16323	2.39189	2.00001	2.00006	1.86755
4(*G*_1_)	1.0	1.55173	1.01042	2.30123	3.19504	1.00000	0.98664
5(*G*_1_)	2.0	1.40069	1.24912	0.32136	0.25317	2.00000	2.01339
6	1.0	1.00000	1.00002	1.00000	1.00000	1.00000	0.98154
7(*G*_2_)	1.0	1.00927	1.02815	1.00000	1.00000	1.00000	0.99124
8(*G*_2_)	1.0	1.32173	0.95504	1.00000	1.00000	1.00000	0.99919
9(*G*_2_)	2.0	1.34185	1.18286	2.00000	2.00000	2.00000	1.93527
10(*G*_2_)	1.0	1.00477	1.01393	1.00000	1.00000	1.00000	0.98693
11	2.0	1.99973	2.00007	2.00000	2.00000	2.00000	2.03582
12	1.0	0.99944	1.00019	1.00000	1.00000	1.00000	1.00435
13(*G*_3_)	1.0	1.00572	1.05126	1.00001	1.00001	1.00001	1.03448
14(*G*_3_)	1.0	1.39147	0.90768	1.00000	1.00000	1.00000	0.99558
15(*G*_3_)	2.0	1.45117	1.00760	2.00003	2.00002	2.00001	1.98699
16(*G*_3_)	1.0	1.00280	1.02531	1.00001	1.00000	1.00001	0.99786
17	2.0	1.99987	1.99999	1.99999	1.99999	1.99999	1.99586
18	1.0	1.00016	1.00000	1.00000	1.00000	1.00000	1.03924
19	0.1	0.10016	0.10000	0.10000	0.10000	0.10000	0.10000
20	1.0	1.00263	1.00000	1.00000	1.00000	1.00001	0.99469
21	0.1	0.10003	0.10000	0.10000	0.10000	0.10000	0.10007
22	0.1	0.10010	0.10000	0.10000	0.10000	0.10000	0.10000
23	1.0	1.00127	1.00000	1.00000	1.00000	1.00000	0.99581
24	0.1	0.10003	0.10000	0.10000	0.10000	0.10000	0.10025
25	0.1	0.10003	0.10000	0.10000	0.10000	0.10000	0.10492
26	1.0	1.00023	1.00002	1.00001	1.00000	1.00001	1.05077
27	0.1	0.10001	0.10000	0.10000	0.10000	0.10000	0.10120
28(*G*_4_)	1.0	0.96519	0.99594	1.00000	1.00000	1.00000	1.01865
29(*G*_4_)	1.0	1.62390	1.04672	1.00000	1.00000	1.00001	0.90507
30(*G*_4_)	1.0	1.56817	1.04245	1.00000	0.99999	1.00000	0.85521
31	1.0	0.99997	1.00000	1.00000	1.00000	1.00000	1.11984
32	1.0	1.00110	1.00000	1.00000	1.00000	1.00000	0.97161
33	1.0	1.00207	0.99998	1.00000	0.99998	0.99998	1.33808
34	1.0	0.99956	1.00000	1.00000	1.00000	1.00000	1.01811
35(*G*_5_)	1.0	1.05000	1.00001	1.00000	1.00000	1.00000	1.05077
36(*G*_5_)	1.0	2.03075	0.99999	1.00000	1.00000	1.00000	1.20947
Residual value		3.62E-9	4.26E-9	5.31E-9	6.49E-9	5.35E-9	1.12E-0

P^*^ are the nominal (true) values, **P**^(1)^ the values based on the 1^st^ data set, **P**^(1)+(2)^ based on the 1^st^, 2^nd^ data sets together, **P**^(1)+(2)+(3)^ based on the 1^st^, 2^nd^, and 3^rd^ data sets, **P**^(1)+…+(4)^ based on the 1^st^ to 4^th^ data sets, and **P**^(1)+…+(5)^ based on the 5 data sets, respectively. (w) means results from 10% noises on the data. Correlated parameter groups are highlighted separately.

**Figure 1:**
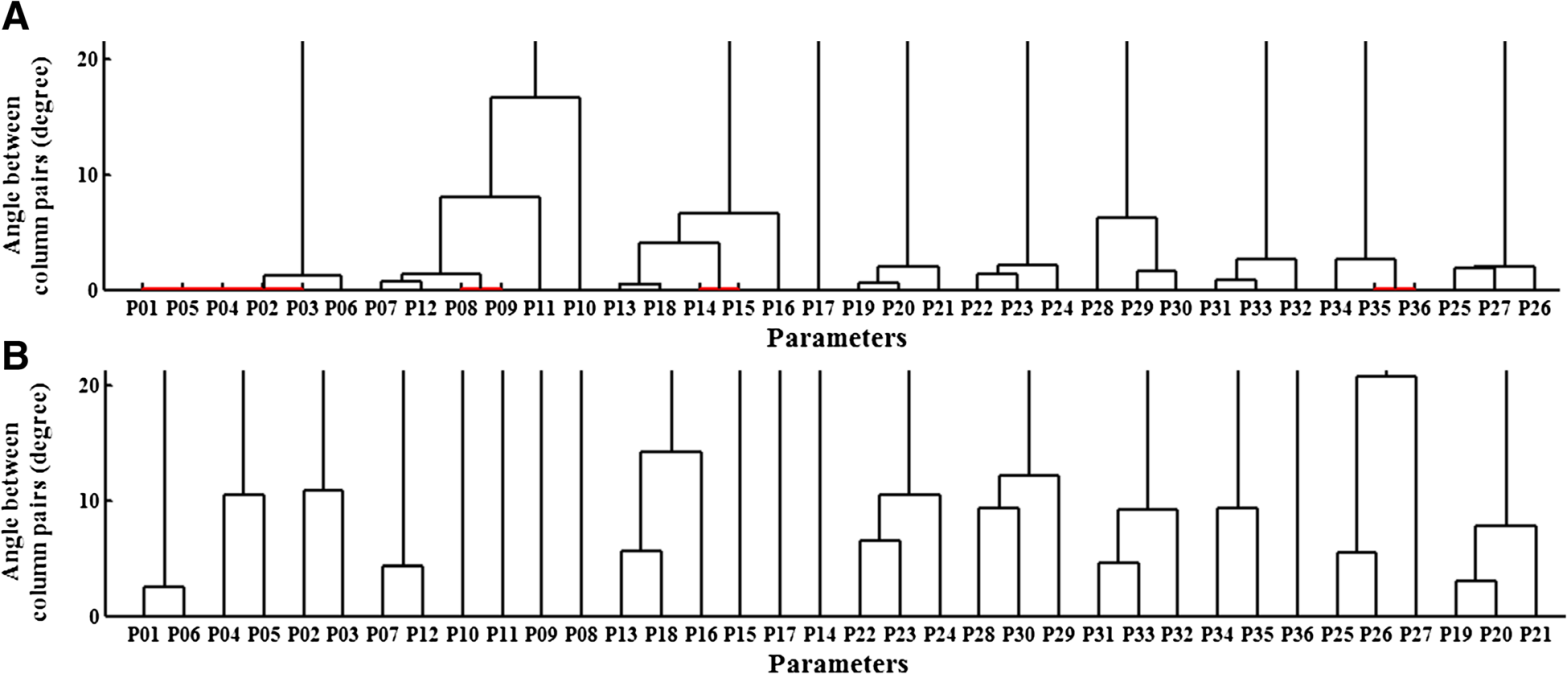
**Dendrogram. (A)** Results from fitting to the 1^st^ data set, where pairwise correlations in different groups exist (red lines). **(B)** Results from fitting to the 5 data sets together, where the pairwise correlations disappear.

Figure 1A (upper panel) shows the angles between the columns of the state sensitivity matrix by fitting to the 1^st^ data set. The zero angles (red lines) mean that the corresponding columns are pairwise parallel. According to Figure 1A, 4 pairwise correlated parameter groups (i.e. (*p*_1_, *p*_2_, *p*_3_, *p*_4_, *p*_5_), (*p*_8_, *p*_9_), (*p*_14_, *p*_15_), (*p*_35_, *p*_36_)) can be detected. However, these are not the same results as identified by the analysis of the model equations. This is because a dendrogram only shows pairwise correlations; it cannot detect higher order interrelationships among the parameters.

**Figure 2:**
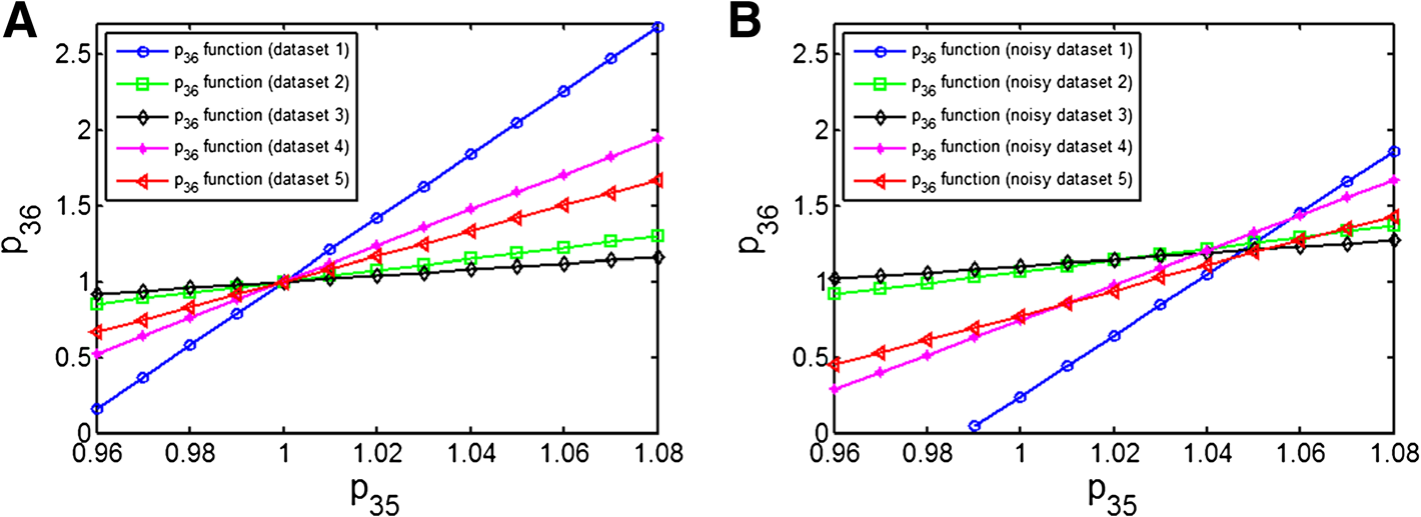
**Correlated relations between *p***_**35 **_**and *p***_**36 **_**based on fitting the model to 5 individual data sets with different inputs. (A)** Fitting to noise-free data sets. The 5 individual zero residual surfaces cross exactly at the true parameter point. It demonstrates that a zero residual surface from any data set will pass through the true parameter point and two data sets will be enough to determine *p*_35_ and *p*_36_. **(B)** Fitting to the data sets with 10% noise. The 5 individual nonzero residual surfaces cross near the true parameter point.

To illustrate the geometric interpretation, we first take the group of *G*_5_(*p*_35_, *p*_36_) as an example to construct ZREs, i.e. to plot the correlated relations between *p*_35_ and *p*_36_. This was done by repeatedly fitting the model to the 5 individual data sets with different inputs, respectively, with fixed values of *p*_35_. The resulting 5 zero residual surfaces (lines) in the subspace of *p*_35_ and *p*_36_ are shown in Figure 2A. As expected, the zero residual surfaces resulted from different data sets cross indeed at the true parameter point in the parameter subspace. Figure 2B shows the relations between *p*_35_ and *p*_36_ by fitting the parameters separately to the same 5 data sets on which a Gaussian distributed error of 10% was added. It can be seen that, due to the measurement noises, the crossing points of the nonzero residual surfaces are at different positions but near the true parameter point. Moreover, by comparing the lines in Figure 2A with Figure 2B, it can be seen that the corresponding zero residual surfaces and nonzero residual surfaces are indeed parallel, when fitting the same data set without noises or with noises, respectively.

**Figure 3:**
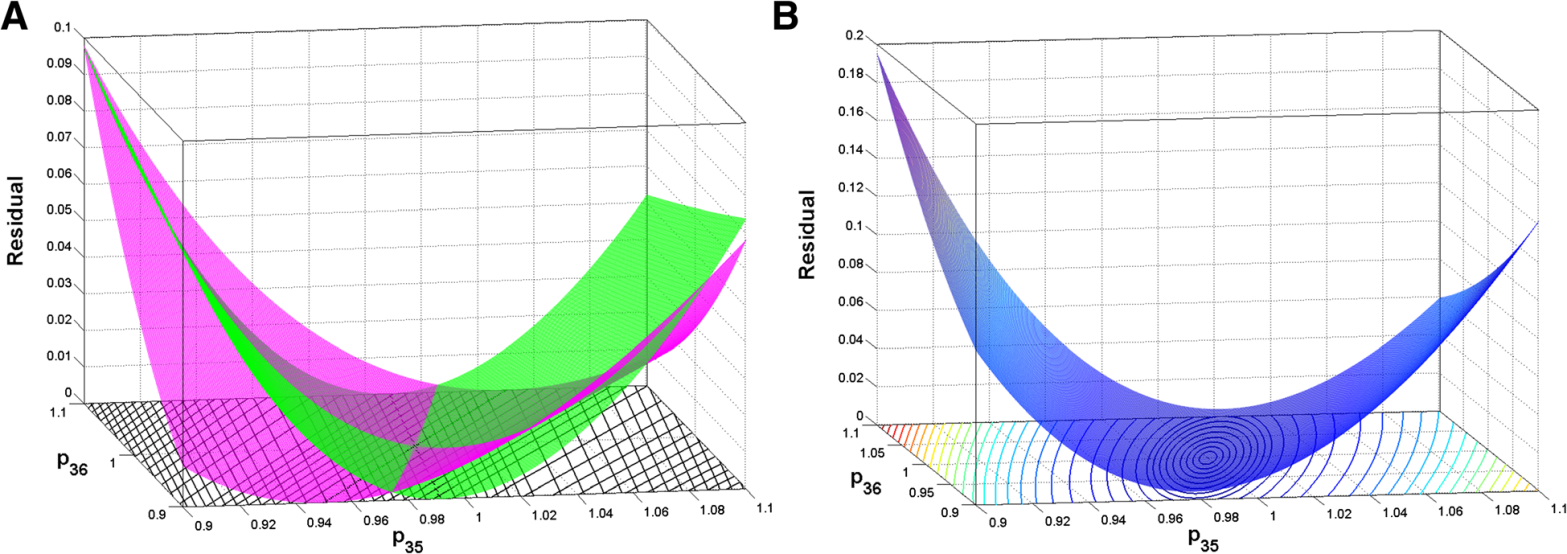
**Residual surfaces of residual values as functions of *p***_**35 **_**and *p***_**36**_**. (A)** Fitting to 2 individual noise-free data sets. **(B)** Fitting to the same 2 data sets together. The true parameter point corresponds to the crossing point in **(A)** and the minimum point in **(B)**.

Figure 3 shows the residual surfaces based on fitting to 2 individual noise-free data sets (Figure 3A) and to the same 2 data sets together (Figure 3B). It is shown from Figure 3A that, due to the correlation, two hyperbolic cylinders are built by separately fitting to individual data sets. The bottom minimum lines of the two cylinders corresponding to the zero residual value cross at the true parameter point. Fitting to the two data sets together leads to an elliptic paraboloid (Figure 3B) which has only one minimum point with the zero residual value. This point is the true parameter point, which means the remedy of the correlation between *p*_35_ and *p*_36_.

Since the maximum number of parameters among the correlation groups is 5, according to our approach, at least 5 data sets with different inputs are needed to uniquely determine the parameter set. The last column in Table 1 (**P**^(1)+…+(5)^) shows the parameter values from fitting the model to the 5 data sets together. It can be seen that all of the 36 parameter values fitted are almost at their true values. According our geometric interpretation, this means that the 5 zero residual surfaces expanded by together fitting to the 5 data sets cross at the true parameter point in the parameter subspace. Figure 1B (lower panel) shows these correlated relations indeed disappear based on the results of fitting to the 5 data sets together.

Moreover, it is shown in Table 1 (**P**^(1)+(2)^) that the correlation between *p*_35_ and *p*_36_ can be remedied by fitting two data sets together. As expected, it can be seen that in **P**^(1)+(2)^ the parameters in *G*_1_ are not well fitted (i.e. 5 correlated parameters cannot be uniquely determined by two data sets). It is also interesting to see in **P**^(1)+(2)^ the parameter values in *G*_2_, *G*_3_ and *G*_4_ are also not well estimated. This is because the degree of freedom of *G*_2_(*p*_7_, *p*_8_, *p*_9_, *p*_10_), *G*_3_(*p*_13_, *p*_14_, *p*_15_, *p*_16_), and *G*_4_(*p*_28_, *p*_29_, *p*_30_) is 3. Indeed, as shown in Table 1 (**P**^(1)+(2)+(3)^), these parameters are exactly determined based on fitting the model to 3 data sets together. However, it is shown in Table 1 from the parameter values of **P**^(1)+(2)+(3)^ and **P**^(1)+…+(4)^ that a number of data sets less than 5 is not enough to remedy the correlations of the parameters in *G*_1_.

To test the sensitivity of the parameter results to measurement errors, we also fitted the model to the same 5 data sets with different inputs and with 10% noise level together. As shown in the last column in Table 1 (**P**^(1)+…+(5)^(w)), to some extent, the parameter values identified are deviated from the true values due to an increased residual value. But the overall parameter quality is quite good. It means the crossing points of the 5 nonzero residual surfaces expanded by the 5 noisy data sets are quite close to the true parameter point.

**Figure 4:**
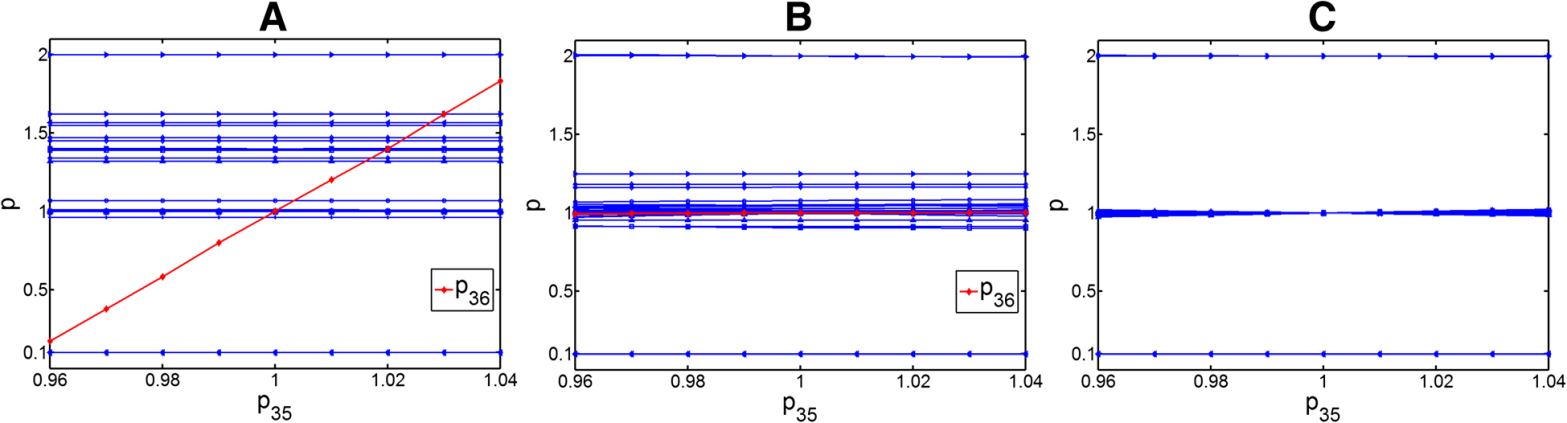
**Relationships of *p***_**35 **_**with other parameters by fitting to different numbers of noise-free data sets with different inputs. (A)** Relations between *p*_35_ and other parameters based on fitting to the 1^st^ data set. **(B)** Relations between *p*_35_ and other parameters based on fitting to 1^st^ and 2^nd^ data sets together. **(C)** Relations between *p*_35_ and other parameters based on fitting to 5 data sets together.

Figure 4 shows profiles of all parameters as a function of *p*_35_, based on different number of data sets used for fitting. It is seen from Figure 4A that only *p*_36_ is correlated with *p*_35_ (red line). Moreover, it can be seen that, by fitting to one data set, the other parameters which have higher order interrelationships in other groups cannot be well determined. As shown in Figure 4B, the correlation between *p*_35_ and *p*_36_ is remedied by fitting to two data sets together and, moreover, the parameters tend to approach their true values (i.e. 0.1, 1.0 and 2.0, see Table 1). Finally, all parameters are uniquely determined (i.e. clearly at the three true values), when 5 data sets were used together for fitting the model, as shown in Figure 4C.

These results clearly demonstrate the scope of our approach to identifying parameter correlations. Moreover, it is clearly seen that adding more data sets with different inputs can remedy the parameter non-identifiability problem in some complex models, but a necessary number of data sets with different inputs (5 for this example) is enough.

**Figure 5:**
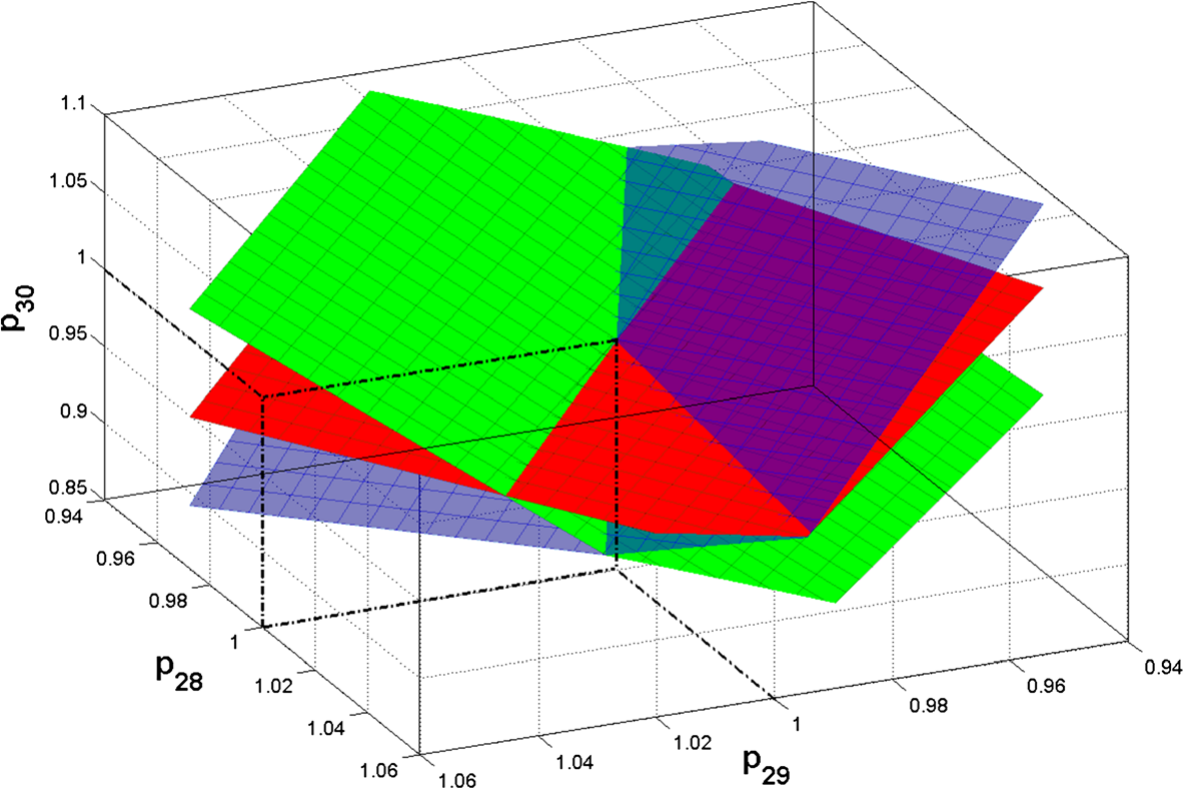
**Relations between *p***_**28**_**, *p***_**29 **_**and *p***_**30 **_**based on fitting the model to 3 individual noise-free data sets with different inputs.** The fittings for *p*_30_ to each data set were made by fixed *p*_28_ and *p*_29_ with different values. Three zero residual surfaces are shown: the green plane is based on 1^st^ data set, the red plane 2^nd^ data set, and the blue plane 3^rd^ data set. The three planes cross exactly at the true parameter point.

To illustrate a higher order interrelationship among parameters, estimations were made by separately fitting the model to 3 individual data sets to plot the relations of the parameters in *G*_4_(*p*_28_, *p*_29_, *p*_30_), as shown in Figure 5. It can be seen that the three zero residual surfaces (planes) resulted from the three individual data sets cross exactly at the true parameter point in the subspace of the 3 parameters. This demonstrates our geometric interpretation of parameter correlations, i.e. to estimate a group of three correlated parameters at least three distinct data sets with different inputs are needed.

Since parameter correlations determined from the proposed approach are based on the structure of the state equations, our result provides a minimum number of different data sets with different inputs necessary for unique parameter estimation (5 in this example). This is definitely true, if all state variables (8 in this example) are measurable and included in the 5 data sets.

The results shown above are from the solutions of the parameter estimation problem based on the data sets composed of *all* 8 state variables. It is demonstrated that at least 5 data sets with different inputs will be needed to uniquely estimate the 36 parameters. However, our method does not give information on how many state variables which may be fewer than 8 but sufficient to identify the 36 parameters. To achieve this information, we tried to estimate the parameters based on the generated 5 data sets which include fewer measured state variables (as output variables). We checked the identifiability when the 5 data sets consist of data profiles of only a part of the state variables. Computational tests were carried out based on different combinations of the state variables included in the data sets. Table 2 shows the minimum sets of state variables which should be included in the data sets so as to achieve a successful fitting. It can be seen, for instance, the 36 parameters can be uniquely estimated in the case that only the first three state variables (i.e. *x*_1_, *x*_2_, *x*_3_) are included in the 5 data sets. Moreover, the generated data profiles of *x*_7_ and *x*_8_ are also enough for identifying the 36 parameters. Due to insufficient data, estimation runs with fewer numbers of the state variables than listed in Table 2 could not converge, i.e. the parameters will be non-identifiable.

**Table 2:** Measurable variable sets for a successful fitting

**No.**	**Measured variables**
**y**_1_	(*x*_1_, *x*_2_, *x*_3_)
**y**_2_	(*x*_1_, *x*_2_, *x*_6_)
**y**_3_	(*x*_1_, *x*_3_, *x*_5_)
**y**_4_	(*x*_1_, *x*_5_, *x*_6_)
**y**_5_	(*x*_2_, *x*_4_, *x*_6_)
**y**_6_	(*x*_4_, *x*_5_, *x*_6_)
**y**_7_	(*x*_7_, *x*_8_)

Different sets of state variables were used as measurable output variables included in the 5 data sets, respectively. This table shows the groups of a minimum number of state variables used as outputs for the parameter estimation which leads to the convergence to the true parameter point.

## Conclusions

It is well recognized that parameters in many biological models are correlated. Finding the true parameter point remains as a challenge since it is hidden in these correlated relations. In many cases, a direct analysis of parameter correlations based on the output sensitivity matrix depends on experimental design, and the analytical relationship cannot be seen. Instead, we presented a method to analyse parameter correlations based on the matrix of the first order partial derivative functions of state equations which can be analytically derived. In this way, pairwise correlations and higher order interrelationships among the parameters can be detected. The result gives the information about parameter correlations and thus about the identifiability of parameters when all state variables are measurable for fitting the parameters. Since the output sensitivity matrix is a linear transformation of the matrix of first order partial derivative functions, our correlation analysis approach provides a necessary (but not sufficient) condition of parameter identifiability. That is, if there exist parameter correlations, the corresponding parameters are non-identifiable.

In addition, we introduced residual surfaces in the parameter subspace to interpret parameter correlations. Any point on a zero residual surface will result in a zero residual value. The crossing point of multiple zero residual surfaces leads to the true parameter point. Zero residual surfaces correspond to ZREs resulted from noise-free data sets used for fitting the parameters. If the ZREs are linearly independent (i.e. there are no correlations), the model parameters are identifiable, and otherwise they are non-identifiable. If more linearly independent ZREs can be constructed by adding new data sets with different inputs, the parameters are practically non-identifiable, otherwise they are structurally non-identifiable. In the case of practical non-identifiability the true parameter values can be found by together fitting the model to a necessary number of data sets which is the maximum number of parameters among the correlation groups. If the available measured data are from output variables, this should be regarded as the minimum number of data sets with different inputs required for unique parameter estimation. The results of the case study demonstrate the feasibility of our approach.

Moreover, an interesting result of our approach is that parameter correlations are not affected by the initial state. This means that, experimental runs can be conducted with any initial state to obtain the required data sets with different inputs. More interestingly, according to this result, different data sets with different inputs can be gained in one experimental run by changing the values of the control inputs. It is noted that the proposed approach does not address the identifiability issue of the initial states which would be a future research aspect.

The result of identifiable parameters determined by the proposed approach is theoretical. This means that the quality of the available data (the noise level, the length of sampling time, etc.) has an important impact on the identifiability issue. Parameters which are theoretically identifiable may not be identifiable by an estimator due to low quality of the data. Non-identifiability issues caused by relative data are not considered in this paper. In addition, the identification of parameter correlations based on the output equations is not considered in this paper.

## Competing interests

The authors declare that they have no competing interests.

## Author’s contributions

PL developed the methodology, wrote the manuscript and supervised the study. QDV wrote the software and designed the study. Both authors read and approved the final manuscript.

## Supplementary Material

Additional file 1 Derivation of the sensitivity matrix, partial derivative functions of the case study, and the values of control inputs for generating data sets in the case study. Click here for file
