# Supplementary material for: Identification of parameter correlations for parameter estimation in dynamic biological models
Source: BMC Syst Biol. 2013 Sep 22;7:91. doi: 10.1186/1752-0509-7-91 (PMC4015753; doi:10.1186/1752-0509-7-91)
Supplement: Additional file 1 — Derivation of the sensitivity matrix, partial derivative functions of the case study, and the values of control inputs for generating data sets in the case study. [file 1752-0509-7-91-S1.pdf]

Additional file 1 for

Identification of parameter correlations for parameter estimation in dynamic biological models

Derivation of the sensitivity matrix, partial derivative functions of the case study, and the values of control inputs for generating data sets in the case study.

1) The sensitivity matrix derivation

Consider the sensitivity equation

$$\dot{S} = \left(\frac{\partial f}{\partial x}\right) S + \left(\frac{\partial f}{\partial p}\right) \quad \text{(A1)}$$

Using the explicit Euler method at time point $t = k\,\Delta t$ with a small time interval Δt , we can write Eq. (A1) in the discrete form

$$\frac{S_k - S_{k-1}}{\Delta t} = \left(\frac{\partial f}{\partial x}\right)_{k-1} S_{k-1} + \left(\frac{\partial f}{\partial p}\right)_{k-1} \quad \text{(A2)}$$

It leads to

$$S_k = S_{k-1} + \Delta t \left(\frac{\partial f}{\partial x}\right)_{k-1} S_{k-1} + \Delta t \left(\frac{\partial f}{\partial p}\right)_{k-1} = \left(I + \Delta t \left(\frac{\partial f}{\partial x}\right)_{k-1}\right) S_{k-1} + \Delta t \left(\frac{\partial f}{\partial p}\right)_{k-1} \quad \text{(A3)}$$

where I is a unit matrix. By expanding Eq. (A3) we get

$$\begin{aligned} S_k = {} & \left(\prod_{i=0}^{k-1}\left(I + \Delta t \left(\frac{\partial f}{\partial x}\right)_i\right)\right) S_0 + \Delta t \left(\prod_{i=1}^{k-1}\left(I + \Delta t \left(\frac{\partial f}{\partial x}\right)_i\right)\right)\left(\frac{\partial f}{\partial p}\right)_0 \\ & + \Delta t \left(\prod_{i=2}^{k-1}\left(I + \Delta t \left(\frac{\partial f}{\partial x}\right)_i\right)\right)\left(\frac{\partial f}{\partial p}\right)_1 + \cdots + \Delta t \left(\frac{\partial f}{\partial p}\right)_{k-1} \end{aligned} \quad \text{(A4)}$$

It can be reformulated as

$$S_k = \left(\prod_{i=0}^{k-1} \left(I + \Delta t \left(\frac{\partial f}{\partial x} \right)_i \right) \right) S_0 + W_0 \left(\frac{\partial f}{\partial p} \right)_0 + W_1 \left(\frac{\partial f}{\partial p} \right)_1 + \cdots + W_{k-1} \left(\frac{\partial f}{\partial p} \right)_{k-1}$$
$$= \left(\prod_{i=0}^{k-1} \left(I + \Delta t \left(\frac{\partial f}{\partial x} \right)_i \right) \right) S_0 + \sum_{j=0}^{k-1} W_j \left(\frac{\partial f}{\partial p} \right)_j \tag{A5}$$

Since $S_0 = \left(\frac{\partial x}{\partial p} \right)_0$ is the sensitivity at the initial state $x(t_0) = x_0$, there are two possible cases:

Case 1: $x(t_0) = x_0$ is a steady state. Then

$$S_0 = \left(\frac{\partial x}{\partial p} \right)_0 = \left(\left(\frac{\partial f}{\partial x} \right)_0 \right)^{-1} \left(\frac{\partial f}{\partial p} \right)_0 \tag{A6}$$

Case 2: $x(t_0) = x_0$ is not a steady state. Then we can consider that $x(t_0) = x_0$ is evolved from a steady state $x(-l) = x_{-l}$ at time point $t = -l$. According to Eq. (A5)

$$S_0 = \left(\prod_{i=-l}^{-1} \left(I + \Delta t \left(\frac{\partial f}{\partial x} \right)_i \right) \right) S_{-l} + \sum_{j=-l}^{-1} W_j \left(\frac{\partial f}{\partial p} \right)_j$$
$$= \left(\prod_{i=-l}^{-1} \left(I + \Delta t \left(\frac{\partial f}{\partial x} \right)_i \right) \right) \left(\left(\frac{\partial f}{\partial x} \right)_{-l} \right)^{-1} \left(\frac{\partial f}{\partial p} \right)_{-l} + \sum_{j=-l}^{-1} W_j \left(\frac{\partial f}{\partial p} \right)_j \tag{A8}$$
$$= \tilde{W}_{-l} \left(\frac{\partial f}{\partial p} \right)_{-l} + \sum_{j=-l}^{-1} W_j \left(\frac{\partial f}{\partial p} \right)_j$$

In both cases, S_0 has a linear relation with $\left(\frac{\partial f}{\partial p} \right)_j$. Then from Eq. (A5) there is

$$S_k = \left(\frac{\partial x}{\partial p} \right)_k = \sum_{j=0}^{k-1} V_j \Delta t \left(\frac{\partial f}{\partial p} \right)_j \tag{A9}$$

where V_j is a matrix computed at the discrete time point *j*. From Eq. (A9), for $\Delta t \to 0$, the sensitivity matrix can be expressed as

$$S = \int_{t_0}^{t} \left(V(\tau) \left(\frac{\partial f}{\partial p} \right) \right) d\tau \tag{A10}$$

2) The partial derivative functions of the three-step-pathway model

According to Eqs. (14-21) in the paper the functions to be partially derived are

$$f_1 = \frac{p_1}{1 + \left(\frac{P}{p_2}\right)^{p_3} + \left(\frac{p_4}{S}\right)^{p_5}} - p_6 x_1 \tag{B1}$$

$$f_2 = \frac{p_7}{1 + \left(\frac{P}{p_8}\right)^{p_9} + \left(\frac{p_{10}}{x_7}\right)^{p_{11}}} - p_{12} x_2 \tag{B2}$$

$$f_3 = \frac{p_{13}}{1 + \left(\frac{P}{p_{14}}\right)^{p_{15}} + \left(\frac{p_{16}}{x_8}\right)^{p_{17}}} - p_{18} x_3 \tag{B3}$$

$$f_4 = \frac{p_{19} x_1}{p_{20} + x_1} - p_{21} x_4 \tag{B4}$$

$$f_5 = \frac{p_{22} x_2}{p_{23} + x_2} - p_{24} x_5 \tag{B5}$$

$$f_6 = \frac{p_{25} x_3}{p_{26} + x_3} - p_{27} x_6 \tag{B6}$$

$$f_7 = \frac{p_{28} x_4 (S - x_7)}{p_{29}\left(1 + \frac{S}{p_{29}} + \frac{x_7}{p_{30}}\right)} - \frac{p_{31} x_5 (x_7 - x_8)}{p_{32}\left(1 + \frac{x_7}{p_{32}} + \frac{x_8}{p_{33}}\right)} \tag{B7}$$

$$f_8 = \frac{p_{31} x_5 (x_7 - x_8)}{p_{32}\left(1 + \frac{x_7}{p_{32}} + \frac{x_8}{p_{33}}\right)} - \frac{p_{34} x_6 (x_8 - P)}{p_{35}\left(1 + \frac{x_8}{p_{35}} + \frac{P}{p_{36}}\right)} \tag{B8}$$

From Eq. (B1),

$$\frac{\partial f_1}{\partial p_1} = \frac{1+\left(\frac{P}{p_2}\right)^{p_3}+\left(\frac{p_4}{S}\right)^{p_5}}{\left[1+\left(\frac{P}{p_2}\right)^{p_3}+\left(\frac{p_4}{S}\right)^{p_5}\right]^2} \tag{B9}$$

$$\frac{\partial f_1}{\partial p_2} = \frac{\frac{p_1 p_3}{p_2}\left(\frac{P}{p_2}\right)^{p_3}}{\left[1+\left(\frac{P}{p_2}\right)^{p_3}+\left(\frac{p_4}{S}\right)^{p_5}\right]^2} \tag{B10}$$

$$\frac{\partial f_1}{\partial p_3} = \frac{-p_1\left(\frac{P}{p_2}\right)^{p_3}\ln\left(\frac{P}{p_2}\right)}{\left[1+\left(\frac{P}{p_2}\right)^{p_3}+\left(\frac{p_4}{S}\right)^{p_5}\right]^2} \tag{B11}$$

$$\frac{\partial f_1}{\partial p_4} = \frac{-\frac{p_1 p_5}{p_4}\left(\frac{p_4}{S}\right)^{p_5}}{\left[1+\left(\frac{P}{p_2}\right)^{p_3}+\left(\frac{p_4}{S}\right)^{p_5}\right]^2} \tag{B12}$$

$$\frac{\partial f_1}{\partial p_5} = \frac{-p_1\left(\frac{p_4}{S}\right)^{p_5}\ln\left(\frac{p_4}{S}\right)}{\left[1+\left(\frac{P}{p_2}\right)^{p_3}+\left(\frac{p_4}{S}\right)^{p_5}\right]^2} \tag{B13}$$

$$\frac{\partial f_1}{\partial p_6} = -x_1 \tag{B14}$$

It can be clearly seen from Eqs. (B9-B13) that these partial derivative functions depend only on the parameters and controls. Thus $\frac{\partial f_1}{\partial p_1}, \frac{\partial f_1}{\partial p_2}, \cdots, \frac{\partial f_1}{\partial p_5}$ are pairwise linearly dependent. From Eq. (B14), $\frac{\partial f_1}{\partial p_6}$ depends on a state variable which will be a time-dependent profile and thus is linearly independent with the other partial derivative functions.

From Eq. (B2),

$$\frac{\partial f_2}{\partial p_7} = \frac{1+\left(\frac{P}{p_8}\right)^{p_9}+\left(\frac{p_{10}}{x_7}\right)^{p_{11}}}{\left[1+\left(\frac{P}{p_8}\right)^{p_9}+\left(\frac{p_{10}}{x_7}\right)^{p_{11}}\right]^2} \tag{B15}$$

$$\frac{\partial f_2}{\partial p_8} = \frac{\frac{p_7 p_9}{p_8}\left(\frac{P}{p_8}\right)^{p_9}}{\left[1+\left(\frac{P}{p_8}\right)^{p_9}+\left(\frac{p_{10}}{x_7}\right)^{p_{11}}\right]^2} \tag{B16}$$

$$\frac{\partial f_2}{\partial p_9} = \frac{-p_7\left(\frac{P}{p_8}\right)^{p_9}\ln\left(\frac{P}{p_8}\right)}{\left[1+\left(\frac{P}{p_8}\right)^{p_9}+\left(\frac{p_{10}}{x_7}\right)^{p_{11}}\right]^2} \tag{B17}$$

$$\frac{\partial f_2}{\partial p_{10}} = \frac{-\frac{p_7 p_{11}}{p_{10}}\left(\frac{p_{10}}{x_7}\right)^{p_{11}}}{\left[1+\left(\frac{P}{p_8}\right)^{p_9}+\left(\frac{p_{10}}{x_7}\right)^{p_{11}}\right]^2} \tag{B18}$$

$$\frac{\partial f_2}{\partial p_{11}} = \frac{-p_7\left(\frac{p_{10}}{x_7}\right)^{p_{11}}\ln\left(\frac{p_{10}}{x_7}\right)}{\left[1+\left(\frac{P}{p_8}\right)^{p_9}+\left(\frac{p_{10}}{x_7}\right)^{p_{11}}\right]^2} \tag{B19}$$

$$\frac{\partial f_2}{\partial p_{12}} = -x_2 \tag{B20}$$

Based on Eqs. (B16-B17), we have

$$\frac{\partial f_2}{\partial p_8} = \left[\frac{-\frac{p_9}{p_8}}{\ln\left(\frac{P}{p_8}\right)}\right]\frac{\partial f_2}{\partial p_9} \tag{B21}$$

Since the coefficient in Eq. (B21) only depends on parameters and a control variable *P*, $\frac{\partial f_2}{\partial p_8}, \frac{\partial f_2}{\partial p_9}$ are linearly dependent. From Eqs. (B15-B18) it can be seen that

$$\frac{\partial f_2}{\partial p_7} - \left[\frac{1+\left(\frac{P}{p_8}\right)^{p_9}}{\frac{p_7 p_9}{p_8}\left(\frac{P}{p_8}\right)^{p_9}} \right] \frac{\partial f_2}{\partial p_8} + \left(\frac{p_{10} p_{11}}{p_7}\right) \frac{\partial f_2}{\partial p_{10}} = 0 \tag{B22}$$

$$\frac{\partial f_2}{\partial p_7} + \left[\frac{1+\left(\frac{P}{p_8}\right)^{p_9}}{p_7 \left(\frac{P}{p_8}\right)^{p_9} \ln\left(\frac{P}{p_8}\right)} \right] \frac{\partial f_2}{\partial p_9} + \left(\frac{p_{10} p_{11}}{p_7}\right) \frac{\partial f_2}{\partial p_{10}} = 0 \tag{B23}$$

Again, the coefficients in Eqs. (B22-B23) only depend on the parameters and the control variable *P*, therefore, two different groups, $\frac{\partial f_2}{\partial p_7}, \frac{\partial f_2}{\partial p_8}, \frac{\partial f_2}{\partial p_{10}}$ and $\frac{\partial f_2}{\partial p_7}, \frac{\partial f_2}{\partial p_9}, \frac{\partial f_2}{\partial p_{10}}$ are linearly dependent, respectively. Similarly, according to Eqs. (B19-B20), $\frac{\partial f_2}{\partial p_{11}}, \frac{\partial f_2}{\partial p_{12}}$ are different from the other partial derivative functions and thus linearly independent with each other and also with other partial derivative functions.

Similar results can be obtained by comparing the partial derivative functions of Eq. (B3), since Eq. (B3) has the similar structure as Eq. (B2). Therefore, $\frac{\partial f_3}{\partial p_{14}}, \frac{\partial f_3}{\partial p_{15}}$ are linearly dependent in pair, $\frac{\partial f_3}{\partial p_{13}}, \frac{\partial f_3}{\partial p_{14}}, \frac{\partial f_3}{\partial p_{16}}$ and $\frac{\partial f_3}{\partial p_{13}}, \frac{\partial f_3}{\partial p_{15}}, \frac{\partial f_3}{\partial p_{16}}$ are linearly dependent in two groups, respectively.

From Eq. (B4),

$$\frac{\partial f_4}{\partial p_{19}} = \frac{x_1}{p_{20} + x_1} \tag{B24}$$

$$\frac{\partial f_4}{\partial p_{20}} = \frac{-p_{19}x_1}{(p_{20}+x_1)^2} \tag{B25}$$

$$\frac{\partial f_4}{\partial p_{20}} = -x_4 \tag{B26}$$

It can be clearly seen that $\frac{\partial f_4}{\partial p_{19}}, \frac{\partial f_4}{\partial p_{20}}, \frac{\partial f_4}{\partial p_{21}}$ are linearly independent. Similarly, according to Eqs. (B5-B6), there are no linear dependences among $\frac{\partial f_5}{\partial p_{22}}, \frac{\partial f_5}{\partial p_{23}}, \frac{\partial f_5}{\partial p_{24}}$ and $\frac{\partial f_6}{\partial p_{25}}, \frac{\partial f_6}{\partial p_{26}}, \frac{\partial f_6}{\partial p_{27}}$.

From Eq. (B7),

$$\frac{\partial f_7}{\partial p_{28}} = \frac{\frac{x_4(S-x_7)}{p_{29}}\left(1+\frac{S}{p_{29}}+\frac{x_7}{p_{30}}\right)}{\left(1+\frac{S}{p_{29}}+\frac{x_7}{p_{30}}\right)^2} \tag{B27}$$

$$\frac{\partial f_7}{\partial p_{29}} = \frac{-\frac{p_{28}x_4(S-x_7)}{p_{29}^2}\left(1+\frac{x_7}{p_{30}}\right)}{\left(1+\frac{S}{p_{29}}+\frac{x_7}{p_{30}}\right)^2} \tag{B28}$$

$$\frac{\partial f_7}{\partial p_{30}} = \frac{\frac{p_{28}x_4(S-x_7)}{p_{29}p_{30}}\left(\frac{x_7}{p_{30}}\right)}{\left(1+\frac{S}{p_{29}}+\frac{x_7}{p_{30}}\right)^2} \tag{B29}$$

$$\frac{\partial f_7}{\partial p_{31}} = \frac{-\frac{x_5(x_7-x_8)}{p_{32}}\left(1+\frac{x_7}{p_{32}}+\frac{x_8}{p_{33}}\right)}{\left(1+\frac{x_7}{p_{32}}+\frac{x_8}{p_{33}}\right)^2} \tag{B30}$$

$$\frac{\partial f_7}{\partial p_{32}} = \frac{\frac{p_{31}x_5(x_7-x_8)}{p_{32}^2}\left(1+\frac{x_8}{p_{33}}\right)}{\left(1+\frac{x_7}{p_{32}}+\frac{x_8}{p_{33}}\right)^2} \tag{B31}$$

$$\frac{\partial f_7}{\partial p_{33}} = -\frac{\frac{p_{31}x_5(x_7 - x_8)}{p_{32}p_{33}}\left(\frac{x_8}{p_{33}}\right)}{\left(1 + \frac{x_7}{p_{32}} + \frac{x_8}{p_{33}}\right)^2} \tag{B32}$$

From Eqs. (B27-B29), $\frac{\partial f_7}{\partial p_{28}}, \frac{\partial f_7}{\partial p_{29}}, \frac{\partial f_7}{\partial p_{30}}$ are linearly dependent in one group. But $\frac{\partial f_7}{\partial p_{31}}, \frac{\partial f_7}{\partial p_{32}}, \frac{\partial f_7}{\partial p_{33}}$ are linearly independent, based on Eqs. (B30-B32).

From Eq. (B8),

$$\frac{\partial f_8}{\partial p_{34}} = \frac{-\frac{x_6(x_8 - P)}{p_{35}}\left(1 + \frac{x_8}{p_{35}} + \frac{P}{p_{36}}\right)}{\left(1 + \frac{x_8}{p_{35}} + \frac{P}{p_{36}}\right)^2} \tag{B33}$$

$$\frac{\partial f_8}{\partial p_{35}} = \frac{\frac{p_{34}x_6(x_8 - P)}{p_{35}^2}\left(1 + \frac{P}{p_{36}}\right)}{\left(1 + \frac{x_8}{p_{35}} + \frac{P}{p_{36}}\right)^2} \tag{B34}$$

$$\frac{\partial f_8}{\partial p_{36}} = \frac{-\frac{p_{34}x_6(x_8 - P)}{p_{35}p_{36}}\left(\frac{P}{p_{36}}\right)}{\left(1 + \frac{x_8}{p_{35}} + \frac{P}{p_{36}}\right)^2} \tag{B35}$$

It can be seen from Eqs. (B33-B35) that $\frac{\partial f_8}{\partial p_{35}}, \frac{\partial f_8}{\partial p_{36}}$ are linearly dependent, but $\frac{\partial f_8}{\partial p_{34}}$ is linearly independent with $\frac{\partial f_8}{\partial p_{35}}, \frac{\partial f_8}{\partial p_{36}}$.

3) Table A1: *P* and *S* values for generating 5 datasets

Dataset	1	2	3	4	5
P	0.05000	0.36840	1.00000	0.09286	0.13572
S	10.0000	2.15440	0.10000	2.15440	2.15440
